# Alcoholic cirrhosis with Wernicke encephalopathy: Clinical case report

**DOI:** 10.1097/MD.0000000000049998

**Published:** 2026-07-31

**Authors:** Hui Liu, Hao Cai, Decai He

**Affiliations:** a Clinical Trial Center, Liver Failure Therapy and Research Center, Institute of Infectious Diseases, Affiliated Suining Traditional Chinese Medicine Hospital of North Sichuan Medical College, Suining, China; b Center for Digestive Disease Treatment and Research, Affiliated Suining Traditional Chinese Medicine Hospital of North Sichuan Medical College, Suining, China.

**Keywords:** differential diagnosis, hepatic encephalopathy, Wernicke encephalopathy

## Abstract

**Rationale:**

Early differentiation between hepatic encephalopathy and Wernicke encephalopathy (WE) is clinically critical in patients with decompensated alcoholic cirrhosis, as vitamin B1 deficiency can produce overlapping neurological symptoms that obscure the correct diagnosis and delay life-saving treatment.

**Patient concerns:**

A 55-year-old male with a 2-year history of alcoholic cirrhosis was admitted to the hospital due to progressively worsening abdominal distension, diarrhea, and fatigue over the month prior to presentation.

**Diagnoses:**

WE was established based on the classic clinical triad of ocular motor dysfunction, ataxia, and mental confusion, supported by characteristic magnetic resonance imaging findings showing symmetric abnormal signals in the periaqueductal region of the midbrain.

**Interventions:**

Initial management comprised comprehensive ascites therapy addressing diarrhea, hyperlactatemia, hypokalemia, and hypoproteinemia. However, on the third hospital day, the patient acutely developed dysarthria, recent memory loss, hand tremors, unsteady gait, and strabismus, which prompted the immediate administration of high-dose intravenous vitamin B1, supplemented with intramuscular and oral B-complex vitamins.

**Outcomes:**

Following targeted thiamine repletion, the patient’s neurological symptoms showed significant and rapid improvement.

**Lessons:**

To our knowledge, this represents a rare case of WE in a critically ill alcoholic cirrhosis patient complicated by hepatic encephalopathy, in the absence of hepatocellular carcinoma or surgical intervention. This case strongly reinforces the need for clinicians to maintain a high index of suspicion for WE in such complex settings to enable timely diagnosis and intervention.

## 1. Introduction

Wernicke encephalopathy is a clinically severe condition primarily caused by thiamin deficiency, an acute neurological disorder particularly observed in patients with malnutrition or diarrhea-induced absorption impairment and accelerated metabolism.^[1]^ Diagnosis remains challenging, especially in patients with decompensated cirrhosis complicated by hepatic encephalopathy (HE). Delayed diagnosis and treatment of Wernicke encephalopathy may lead to irreversible neurological damage or even death, as timely intervention can significantly improve outcomes and reduce mortality rates.^[2,3]^ The European Federation of Neurological Societies guidelines explicitly identify the diagnostic difficulties of Wernicke’s encephalopathy as a critical challenge that requires urgent resolution.^[4]^ This article presents a critical case report of a patient with alcoholic cirrhosis who developed decompensation and Wernicke’s encephalopathy.

## 2. Consent

This case does not require ethics committee approval as it involves routine clinical practice or well-established procedures that fall under the first category of medical technologies, which are exempt from ethical review according to the relevant regulations. Written informed consent was obtained from the patient for publication of this case, and a copy of the consent form is available for review by the editors of this journal.

## 3. Case report

The patient, a 55-year-old male, was admitted to the hospital with “alcoholic cirrhosis for 20+ years, aggravated with abdominal distension for 20+ days.” He had a drinking habit for over 20 years but did not undergo relevant examination and treatment for economic reasons.

Twenty days before admission, the patient developed abdominal distension, diarrhea, and progressive fatigue. The laboratory test results upon hospital admission are as follows: white blood cell count, 5.5 × 10^9^/L; neutrophil percentage, 67.5%; red blood cell count, 2.4 × 10^12^/L; platelet count, 43 × 10^9^/L; hemoglobin, 69 g/L; super-sensitive C-reactive protein, 26.54 mg/L; blood ammonia concentration,71.0 μmol/L (reference range, 0–30 μmol/L); serum alanine aminotransferase, 43 U/L; serum aspartate aminotransferase, 272 U/L; serum alkaline phosphatase, 224 U/L; serum γ-glutamyl transferase, 1287 U/L; total serum bilirubin, 30 μmol/L (reference range, 3.0–22.0 μmol/L); serum albumin, 32.3 g/L (reference range, 35–50 g/L); serum creatinine, 58.8 μmol/L (reference range, 58–110 μmol/L); serum uric acid, 860 μmol/L (reference range, 208–506 μmol/L); serum potassium, 3.6 mmol/L (reference range, 3.5–5.2 mmol/L); plasma lactate, 5.79 mmol/L (reference range, 0.5–1.6 mmol/L); plasma fibrinogen degradation product, 24.5 μg/mL (reference range, 0–5 μg/mL); plasma D-dimer, 12.58 mg/L (reference range, 0–1.0 mg/L); serum alpha-fetoprotein (AFP), 2.66 ng/mL (reference range, 0–10 ng/mL). Abdominal computed tomography revealed cirrhosis, portal hypertension, ascites, and splenomegaly. The patient declined an endoscopic examination. A neurological examination revealed no abnormalities.

After admission, the patient’s abdominal distension, diarrhea, and progressive fatigue were cured on the third day using piperacillin sodium/tazobactam sodium (8:1 ratio) (4500 mg IV Q8H) combined with Bifidobacterium live bacteria (700 mg PO BID). The symptoms of hypokalemia and hypoproteinemia improved after intravenous fluid therapy, but the serum potassium and albumin levels did not reach the lower limit of normal.

On the second day of hospitalization, the patient’s laboratory test results were as follows: white blood cell count, 5.9 × 10^9^/L; neutrophil percentage, 68.8%; red blood cell count, 2.2 × 10^12^/L; platelet count, 41 × 10^9^/L; hemoglobin, 66 g/L; C-reactive protein (CRP), 24.6 mg/L; serum potassium, 3.23 mmol/L. No changes in mental status were observed.

On the third day of hospitalization, the patient suddenly became unresponsive and lost memory of the recent events. Laboratory test results for the third day were as follows: white blood cell count, 5.6 × 10^9^/L; neutrophil percentage, 56.6%; red blood cell count, 2.2 × 10^12^/L; platelet count, 60 × 10^9^/L; hemoglobin, 65 g/L; blood ammonia, 23.6 µmol/L; serum alanine aminotransferase, 28 U/L; serum aspartate aminotransferase, 100 U/L; serum alkaline phosphatase, 139 U/L; serum γ-glutamyl transferase, 955 U/L; total serum bilirubin, 27.27 mmol/L; serum albumin, 29.1 g/L; serum creatinine, 56.9 mmol/L; serum uric acid, 482 μmol/L; serum potassium, 3.46 mmol/L.

**Figure 1.**
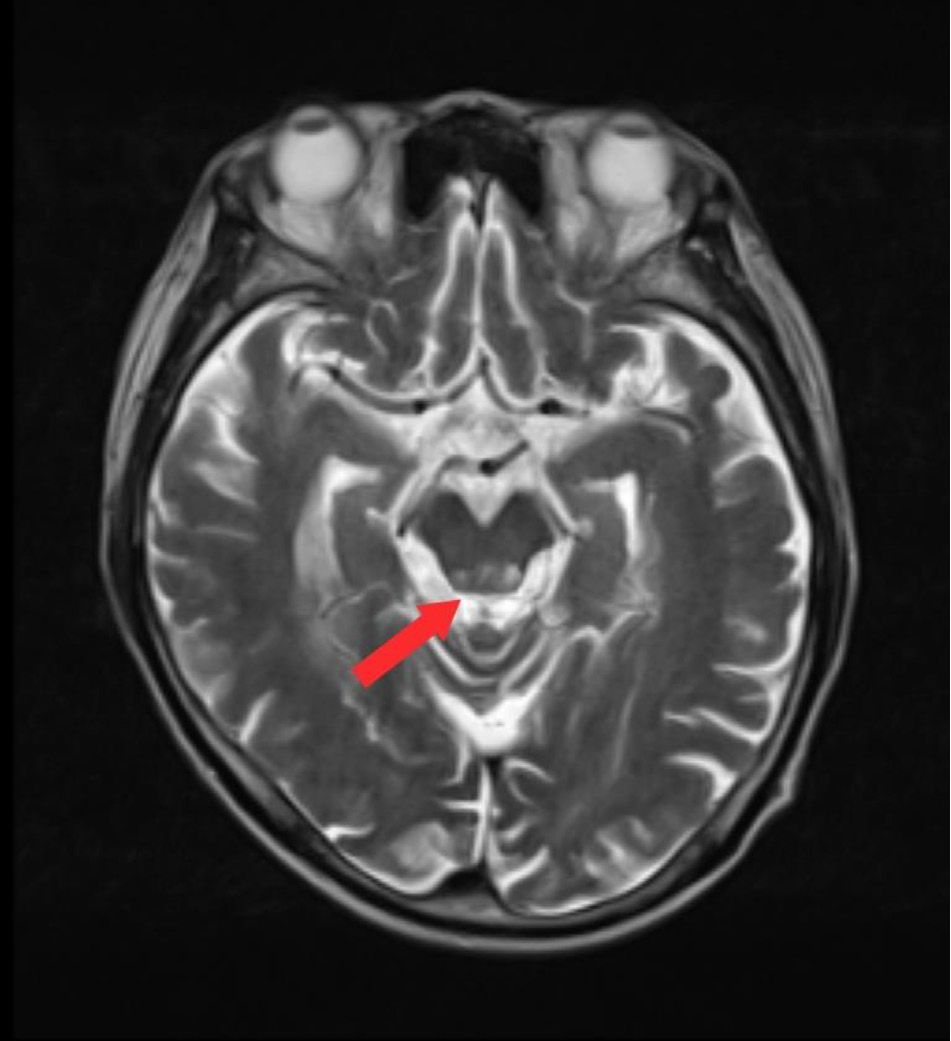
Magnetic resonance imaging manifestation reveals abnormal symmetric signals in the pericentric region of the midbrain, symmetrically distributed patches with slightly prolonged T1 and T2 signals around the bilateral lateral ventricles.

Simultaneously, the patient exhibited symptoms of hand tremors, an unsteady gait, and strabismus, failed to recognize, and experienced language expression impairment. Consequently, the doctor promptly performed a brain magnetic resonance imaging (MRI) scan, which revealed abnormal symmetric signals in the pericentric region of the midbrain, symmetrically distributed patches with slightly prolonged T1 and T2 signals around the bilateral lateral ventricles (Fig. 1). After urgent teleconsultation with neurology specialists and an extensive review of the literature, the patient was ultimately diagnosed with WE based on clinical manifestations and MRI findings. The doctor prescribed a treatment regimen of 100 mg of intravenous vitamin B1 and 200 mg of intramuscular vitamin B1, followed by oral compound vitamin B (containing 20 mg of B1), administered 3 times daily for 5 consecutive days, totaling 1800 mg. Over the following days, the patient’s memory loss, unsteady gait, and strabismus symptoms gradually improved, and by the end of the vitamin B1 course, these symptoms completely resolved.

## 4. Discussion

Wernicke encephalopathy (WE), a clinical emergency, is characterized by a classic triad of symptoms, including confusion of consciousness, ataxia, and ocular muscle paralysis. This condition, often linked to thiamin deficiency, particularly in cases of chronic alcoholism, requires prompt medical intervention to prevent severe complications, with mortality rates ranging from 10 to 20%.^[5]^ Despite the well-documented causes of WE, which include chronic alcoholism and thiamin deficiency, the symptoms are not always apparent, complicating the differential diagnosis and definitive confirmation. Autopsy studies have reported a prevalence of WE ranging from 0.4 to 2.8%, with chronic alcoholism being the primary cause, as most patients with WE have a history of alcohol addiction or other forms of alcohol use.

However, the condition may be overlooked in up to 68% of alcoholic patients, with this figure increasing to 94% in nonalcoholic patients. The Caine diagnostic criteria have been validated as the optimal diagnostic reference, as summarized in Table 1.^[6]^

**Table 1:** Caine criteria for diagnosis of Wernicke encephalopathy.

Possible causes	Signs and symptoms	Auxiliary examination
Dietary deficiencies Gastrointestinal surgery Gastrointestinal tract diseases Vomiting Pancreatitis Psychiatric diseases Other	Altered mental state or mild Memory impairment Cerebellar dysfunction Eye signs Gait ataxia	Magnetic resonance imaging

The diagnosis of WE is primarily clinical and based on the Caine criteria, in which a person must display at least 2 of the 3 classic symptoms or evidence of nutritional deficiency. MRI is a valuable tool for diagnosing WE, as it typically shows symmetric T2/FLAIR hyperintense signals in specific brain regions.

MRI = magnetic resonance imaging, WE = Wernicke encephalopathy.

In this scenario, chronic alcoholism combined with chronic dysregulation of glucose metabolism due to diarrhea jointly led to the development of WE. In emergency scenarios, differentiating HE from WE in patients with underlying alcoholic cirrhosis can be particularly challenging owing to overlapping symptoms and the need for rapid assessment. Alcoholics often exhibit thiamin deficiency owing to inadequate diet, gastrointestinal disturbances, and liver pathology. Furthermore, both alcohol and its metabolite acetaldehyde exert direct toxic effects on thiamin-related enzymes in the liver and brain.^[7]^ Hepatic encephalopathy, a severe complication of liver cirrhosis, is often characterized by elevated ammonia or endorphin levels, resulting in severe psychological disorders, oral health issues, and motor dysfunction. It is a common and potentially life-threatening complication of liver failure, with mortality rates that can be influenced by factors such as disease progression and patient health status.^[8–10]^ Conversely, the most salient symptoms of WE are often mental changes, which can include confusion, apathy, and disorientation. Timely recognition and diagnosis of WE are crucial for the prognosis of this medical emergency. Without timely detection and intervention, this condition can lead to the development of Wernicke–Korsakoff syndrome, which may result in coma or even death. The mortality rate of WE, a component of this syndrome, can reach up to 20% if not properly managed.^[11–14]^ Notably, upon observing early symptoms in patients, physicians should swiftly recognize these as indicators of WE. In fact, it is still difficult to distinguish HE from WE in a timely and accurate manner in clinical practice.^[15]^

First, in patients with decompensated alcoholic cirrhosis, these 2 diseases are associated with psychiatric symptoms. Second, most hepatologists in clinical practice lack familiarity with WE diagnosis. Third, differences in neuroimaging manifestations between the 2 disorders are difficult to distinguish. Similar to the MRI features of WE, some HE patients exhibit symmetrical slightly hyperintense T1 and T2 lesions around the ventricles on MRI, with enhanced FLAIR signals showing deepened sulci and narrowed gyri. The ventricular and cistern systems are enlarged.^[16,17]^ In this case, multiple physicians involved in the discussion initially believed that the patient had HE. However, after observing significant improvement in neurological symptoms following the administration of intravenous and intramuscular vitamin B1 injections combined with oral vitamin B supplementation, they reconsidered their original diagnosis.

Therefore, when differentiating between HE and WE, intravenous infusion of vitamin B1 (100 mg), intramuscular injection of vitamin B1 (200 mg), and oral administration of compound vitamin B (containing 20 mg of B1), totaling 360 mg, may serve as the first-line empirical treatment for suspected WE.^[18]^

To the best of our knowledge, this is a very rare case of WE diagnosed in a patient with advanced cirrhosis, unrelated to hepatocellular carcinoma or surgical procedures, which underscores the potential link between severe liver disease and this neurological condition. Such patients may be overlooked because of the difficulty in distinguishing WE from HE. This case also serves to alert physicians to the risk of sudden-onset WE in patients with alcoholic cirrhosis.

## Acknowledgments

The authors thank the Doctoral Research Start-up Fund of the North Sichuan Medical College (No. CBY24-QDA50) for their support.

## Author contributions

**Conceptualization:** Hui Liu, Hao Cai.

**Funding acquisition:** Hui Liu, Decai He.

**Investigation:** Hui Liu.

**Data curation:** Hui Liu.

**Investigation:** Hui Liu, Hao Cai, Decai He.

**Writing – original draft:** Hui Liu, Hao Cai.
